# The efficacy and safety of Apatinib combined with TACE in the treatment of hepatocellular carcinoma: a meta-analysis

**DOI:** 10.1186/s12957-021-02451-8

**Published:** 2022-03-04

**Authors:** Anan Gong, Xiaofei Li

**Affiliations:** 1 Department of Hepatobiliary Surgery, YiWu Central Hospital, No. 519 Nan men Street, Yiwu, Zhejiang, 322000 China; 2 Department of Infectious Diseases, YiWu Central Hospital, No. 519 Nan men Street, Yiwu, Zhejiang, 322000 China

**Keywords:** Apatinib, TACE, Liver, Cancer, Treatment, Effect, Safety, Review

## Abstract

**Background:**

The timely and effective treatments are vital to the prognosis of patients with hepatocellular carcinoma, and the role of Apatinib combined with TACE in the treatment of hepatocellular carcinoma remains unclear. Therefore, we aimed to conduct a systematic review and meta-analysis to evaluate the efficacy and safety of Apatinib combined with transcatheter arterial chemoembolization (TACE) in the treatment of hepatocellular carcinoma.

**Methods:**

We searched for randomized controlled trials (RCTs) on Apatinib and TACE use in the treatment of hepatocellular carcinoma. Cochrane Central Register of Controlled Trials, Embase, PubMed, China Biomedical Literature Database, China Knowledge Network, Wanfang Database, and Weipu Chinese Science and Technology Journal Database were searched up to 16 April 2021. Two researchers independently screened the literature and extracted data according to the inclusion and exclusion criteria. RevMan 5.3 software was used for Meta-analysis. This meta-analysis protocol had been registered online (available at: https://inplasy.com/inplasy-2021-6-0047/).

**Results:**

A total of 14 RCTs involving 936 hepatocellular carcinoma patients were included. The objective remission rate (OR = 2.93, 95% CI 2.17–3.95), 1-year survival (OR = 2.47, 95% CI 1.65–3.68), 2-year survival (OR = 2.67, 95% CI 1.41–5.04), the incidence of hand-foot syndrome (OR = 32.09, 95% CI 10.87–94.74) and the incidence of proteinuria (OR = 14.79, 95% CI 6.07–36.06) of the Apatinib + TACE group was significantly higher than that of the TACE group (all *P* < 0.05). There were no significant differences in the incidence of myelosuppression (OR = 1.01, 95% CI 0.61–1.67), the incidence of hypertension (OR = 7.56, 95% CI 0.95–1.67, *P* = 60.17) between Apatinib + TACE and TACE group (all *P* > 0.05).

**Conclusions:**

Apatinib combined with TACE is more effective than TACE alone in the treatment of hepatocellular carcinoma, but it has certain adverse reactions.

**Supplementary Information:**

The online version contains supplementary material available at 10.1186/s12957-021-02451-8.

## Introduction

At present, hepatocellular carcinoma is the sixth commonly-seen malignant cancer in the world, and its mortality ranks fourth amongst all cancers in the world [1]. In China, the number of new cases of hepatocellular carcinoma accounts for about half of the world, and there are about 1 million new hepatocellular carcinoma patients every year [2]. Meanwhile, the patients with hepatocellular carcinoma are getting younger and younger, which seriously endangers human health [3, 4]. Due to the lack of early diagnosis and treatments, the disease progresses rapidly and the prognosis of patients with hepatocellular carcinoma is usually poor [5]. Hepatocellular carcinoma has become a major cause of cancer-associated death. It is reported that only 20% of patients are eligible for surgical resection, and the long-term effect is not satisfactory, and the 5-year survival rate is not high [6]. Therefore, the early detection and treatment of hepatocellular carcinoma are essential to the prognosis of patients [7].

Transcatheter arterial chemoembolization (TACE) is a non-surgical treatment for advanced hepatocellular carcinoma [8]. TACE has the advantages of small trauma and high targeting, it can significantly inhibit the progression of tumor tissue, and its short-term effect is obvious [9]. TACE treatment of hepatocellular carcinoma creates a nutrient-deficient environment for tumor cells [10]. Chemotherapy drugs and iodized oil embolization emulsion reach the tumor blood vessels, but hypoxia is an important factor that stimulates the growth of vascular endothelial growth factor (VEGF), so hepatocellular carcinoma recurs and metastasis are rapid [11]. It’s been reported that the objective remission rate is only 10–20% after TACE, and cancers are prone to recurrence and metastasis, leading to unsatisfactory therapeutic effects [12, 13]. Therefore, it is necessary to evaluate TACE combined with various other methods to inhibit angiogenesis, in order to improve the curative effect of hepatocellular carcinoma and delay the progression of cancers.

Apatinib is a new type of molecularly targeted anti-angiogenic drug that can selectively inhibit VEGF receptor 2 to inhibit tumor blood vessel growth and produce anti-tumor function [14]. Apatinib has been applied to the treatment of gastric cancer, breast cancer and other cancers [15–17]. In addition, a phase II clinical study of hepatocellular carcinoma has confirmed the efficacy of Apatinib on hepatocellular carcinoma [18]. Studies [19, 20] have reported that Apatinib can significantly improve the objective remission rate of patients with hepatocellular carcinoma, but there is no relevant systematic review on the role of combined use of Apatinib and TACE. Therefore, we aimed to conduct a meta-analysis of the safety and effectiveness of randomized controlled trial (RCTs) on the application of Apatinib and TACE in the treatment of hepatocellular carcinoma, to elucidate the role of Apatinib and TACE use, and to provide evidence for the treatment of hepatocellular carcinoma. We conducted this meta-analysis according to the PICOS principle of the Cochrane Collaboration. The question framework for this meta-analysis was as following. P(patient): patients with hepatocellular carcinoma. I(intervention): application of Apatinib and TACE in the treatment of hepatocellular carcinoma; C(control): application of TACE in the treatment of hepatocellular carcinoma; O(outcomes): objective remission rate, survival rate and related treatment complications; S(study design):RCT. This meta-analysis protocol had been registered online (available at: https://inplasy.com/inplasy-2021-6-0047/) with registered number: INPLASY202160047.

## Methods

### Literature search strategy

We combined the subject word and free word to search the PubMed, Embase, Cochrane library, China Biomedical Literature Database, China Knowledge Network, Wanfang Database, and Weipu Chinese Science and Technology Journal Database. Besides, we searched clinical trials (https://clinicaltrials.gov/) and Chinese Trial Database (http://www.chictr.org.cn/abouten.aspx) for some unpublished data. The search time limit was from the inception to 16 April 16 2021. The search strategies were as following: (“Neoplasms” OR “Hepatic” OR “Neoplasms” OR “Liver” OR “Liver Neoplasm” OR “Neoplasm” OR “Liver” OR “Hepatic Neoplasm” OR “Hepatocellular Cancers”) AND (“Apatinib”) AND (“Transcatheter arterial chemoembolization” OR “TACE” OR “Hepatic arterial chemoembolization”), we revised the strategies according to the characteristics of databases (see the supplementary Table 1 for more details). The language we screened was limited to English and Chinese language in this present meta-analysis.

### Inclusion and exclusion criteria

The inclusion criteria of this study were the type of study design was RCT, and the populations of the study are patients diagnosed with hepatocellular carcinoma by pathological examination. The control group was treated with TACE, and the experimental group was treated with Apatinib in combination with TACE. The dosage and period of Apatinib administration were not limited. Outcome indicators such as objective remission rate, 1-year, 2-year survival, and related treatment complications were reported. The exclusion criteria of the present meta-analysis were literature review articles, animal studies, case reports, and observational studies were excluded. And we excluded the reports that did not provide detailed postoperative indicators, duplicate articles studies involving patients with malignancies other than hepatocellular carcinoma.

### Quality evaluation and bias risk assessment of included studies

Two researchers independently screened the literature and extracted data. If there was any disagreement during the process, discussions were conducted for consensus, and third-party opinions would be sought if necessary. We would contact the corresponding author for missing information. The included RCTs were analyzed according to the Bias Risk Evaluation Tool of Cochrane Handbook for Systematic Reviews [21]. This tool evaluated seven specific domains, including: sequence generation, allocation concealment, blinding of participants and personnel, blinding of outcome assessment, incomplete outcome data, selective outcome reporting, and other issues. Each domain could be classified as low risk of bias, high risk of bias or unclear risk of bias based on related judgment criteria.

### Data collection

Two authors independently collected following information: author name, year of publications, sample size, characteristics of included patients, details of intervention, outcome indicators, and the main conclusions. The primary outcome observed in this meta-analysis was objective remission rate, which was the complete disappearance of all target lesions or the sum of the diameters of all measurable target lesions is lower than the baseline by ≥ 30%. The secondary outcome indicators were 1-year, 2-year survival, and related treatment complications including incidence of hand-foot syndrome, proteinuria, myelosuppression, and hypertension.

### Statistical methods

The related data was extracted and sorted out, and the RevMan 5.3 software was used for meta-analysis. We used *χ*2 test to analyze and evaluate the heterogeneity of the results. If there was no heterogeneity between the data of each group (*P* > 0.1, *I*^2^ < 50%), then we used the fixed effects model to analyze the data; If the heterogeneity was significant (*P* ≤ 0.1, *I*^2^ ≥ 50%), we firstly identified the potential source of the heterogeneity, and after excluding the influence of obvious clinical heterogeneity, a random effects model was used for meta-analysis. Publication bias was evaluated by using funnel plots, and asymmetry was assessed by conducting Egger regression test. For funnel plot asymmetry, *P*  <  .1 was considered as significantly different.

## Results

### Literature search results

**Fig. 1:**
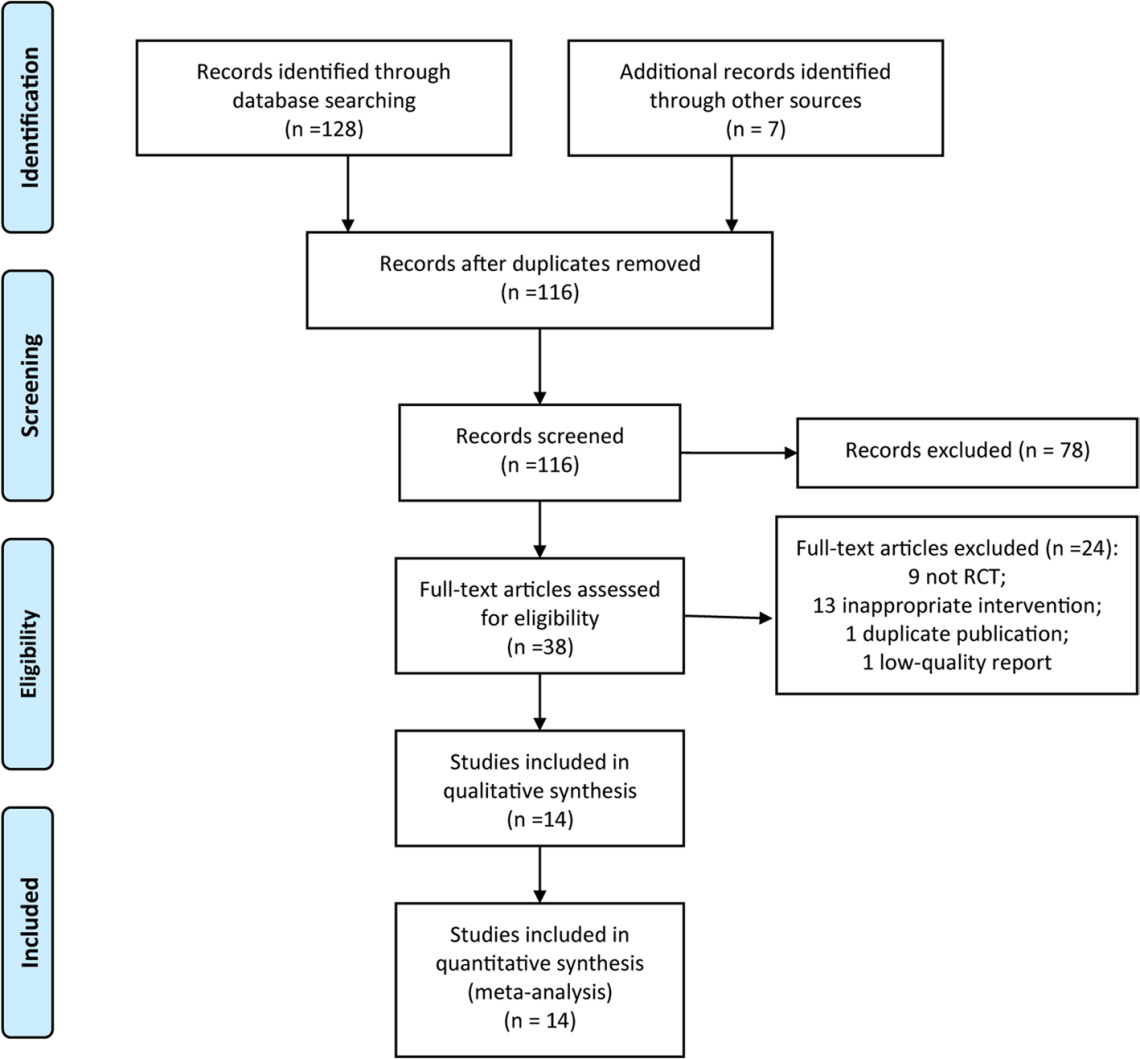
PRISMA flow diagram for study selection

A total of 116 reports were retrieved from the initial search, and after screening layer by layer, 14 RCTs [22–35] were finally included. The study selection process was indicated in Fig. 1.

### The characteristics of included RCTs

Amongst the 14 included RCTs, a total of 936 patients with hepatocellular carcinoma were involved, with 463 patients received Apatinib and TACE treatment, 473 patients received TACE treatment. As presented in Table 1, all the included studies were reported from China. The dose of Apatinib ranged from 250 mg/day to 850 mg/day amongst RCTs.

**Table 1:** The characteristics of included studies

Studies	Cases		Type of hepatocellular carcinoma	Stage of hepatocellular carcinoma(Edmonson tumor grades)	Age		Intervention		
	Apatinib +TACE group	TACE group			Apatinib +TACE group	TACE group	Apatinib +TACE group	TACE group	Durations of intervention(months)
Bai 2018 [36]	25	25	SHCC	II~III	58.34±5.67	59.22±5.17	Apatinib 500 mg/d +TACE group	TACE	3
Jin 2017 [37]	20	22	SHCC	II~IV	55.24±1 0.64	54.12±1 1.48	Apatinib 500 mg/d +TACE group	TACE	3
Li 2018 [38]	54	52	SHCC	II~III	53.93±5.12	55.20±5.25	Apatinib 500 mg/d +TACE group	TACE	6
Lu 2019 [39]	22	21	SHCC	II~III	58.93±9.38	56.41±10.79	Apatinib 500 mg/d +TACE group	TACE	3
Wang 2017 [40]	43	43	SHCC	II~III	58.28±5.21	58.29±5.22	Apatinib 500 mg/d +TACE group	TACE	3
Wu 2019 [41]	28	31	SHCC	II~III	55.93±11.04	56.9±10.19	Apatinib 500 mg/d +TACE group	TACE	3
Zeng 2018 [42]	38	38	SHCC	NA	56.26±4.18	56.48±3.85	Apatinib 850 mg/d +TACE group	TACE	3
Huang 2018 [43]	30	30	SHCC	I~III	52.45±9.12	52.22±9.47	Apatinib 850 mg/d +TACE group	TACE	6
Xie 2019 [44]	42	50	SHCC	NA	53.56±9.16	52.98±9.24	Apatinib 250 mg/d +TACE group	TACE	3
Cui 2019 [45]	25	25	SHCC	II~IIV	51.62±9.64	52.24±9.88	Apatinib 500 mg/d +TACE group	TACE	3
Huang 2017 [46]	38	38	SHCC	II~III	53.09±10.42	52.91±9.26	Apatinib 850 mg/d +TACE group	TACE	3
Wu 2018 [47]	28	28	SHCC	II~III	52.11±10.25	52.42±10.71	Apatinib 500 mg/d +TACE group	TACE	3
He 2018 [48]	50	50	SHCC	II~III	52.14±9.17	55.37±10.33	Apatinib 400 mg/d +TACE group	TACE	3
Li 2017 [49]	20	20	SHCC	II~IV	49.17±10.27	51.06±10.12	Apatinib 850 mg/d +TACE group	TACE	3

*SHCC* small hepatocellular carcinoma, *NA* not available, *TACE* transcatheter arterial chemoembolization

### Quality assessment

**Fig. 2:**
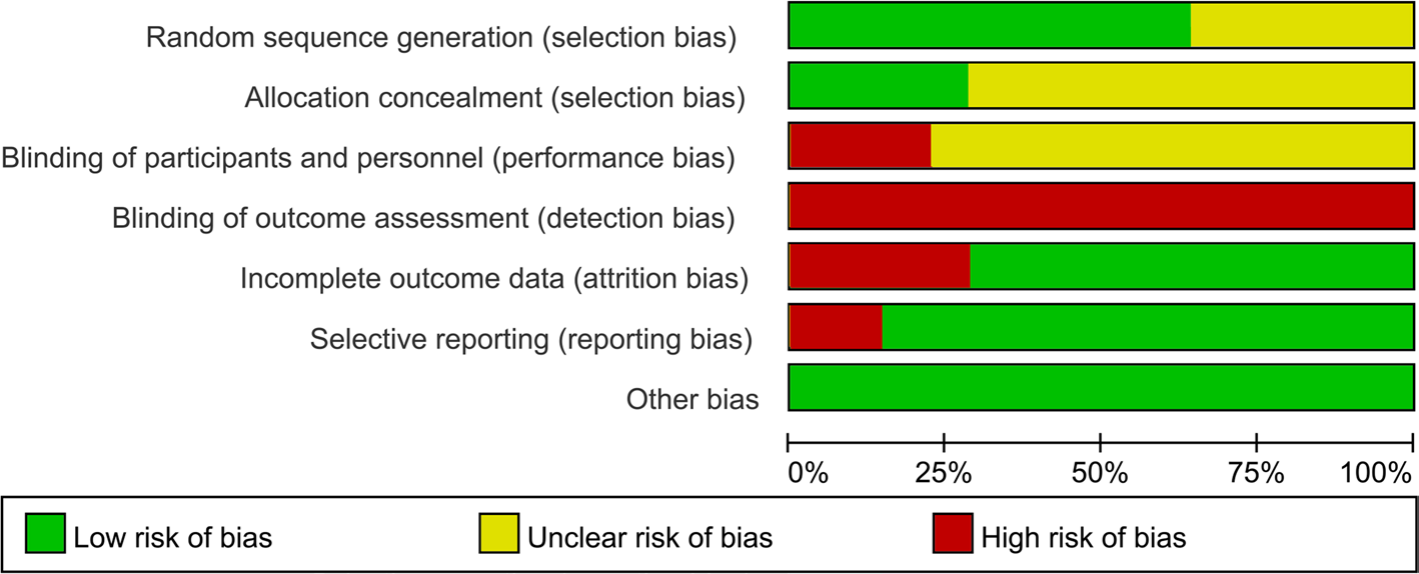
Risk of bias graph

**Fig. 3:**
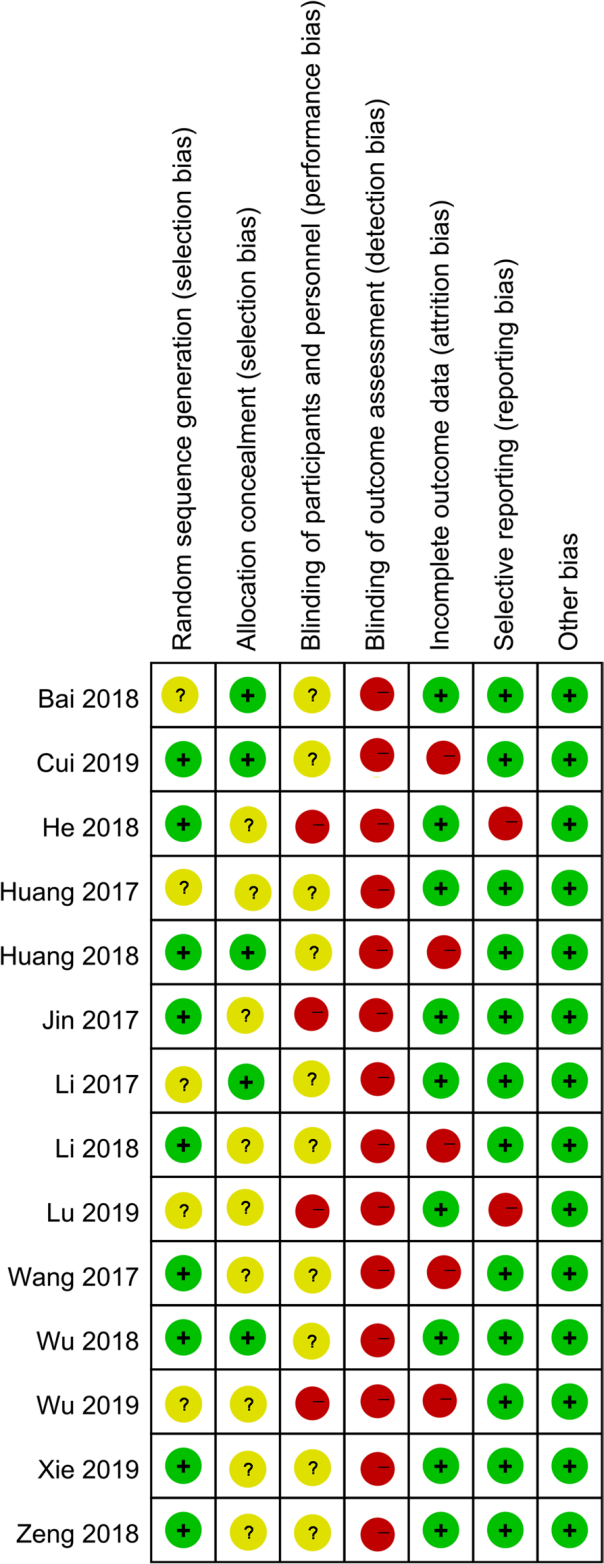
Risk of bias summary

The risk of biases assessments were presented in Figs. 2 and 3. Among the included RCTs, nine RCTs [23, 25–27, 29–31, 33, 35] clearly stated the use of random number table method to generate the randomization (low risk), and five RCTs [22, 24, 26, 28, 32] mentioned the use of allocation concealment. For the blinding method, only four RCTs [23, 24, 33, 34] were clearly single-blind (high risk), and the rest did not clearly state the setting of the blinding method. None of the included RCTs clearly stated their blinding design on the outcome assessment. Five RCTs [26, 27, 29, 31, 35] had been rated as attrition biased with regard to the small samples and loss of follow-up of included patients. Two RCTs [23, 34] selectively reported the outcomes. No significant biases in other biases were found.

### Meta-analysis

#### Objective remission rate

**Fig. 4:**
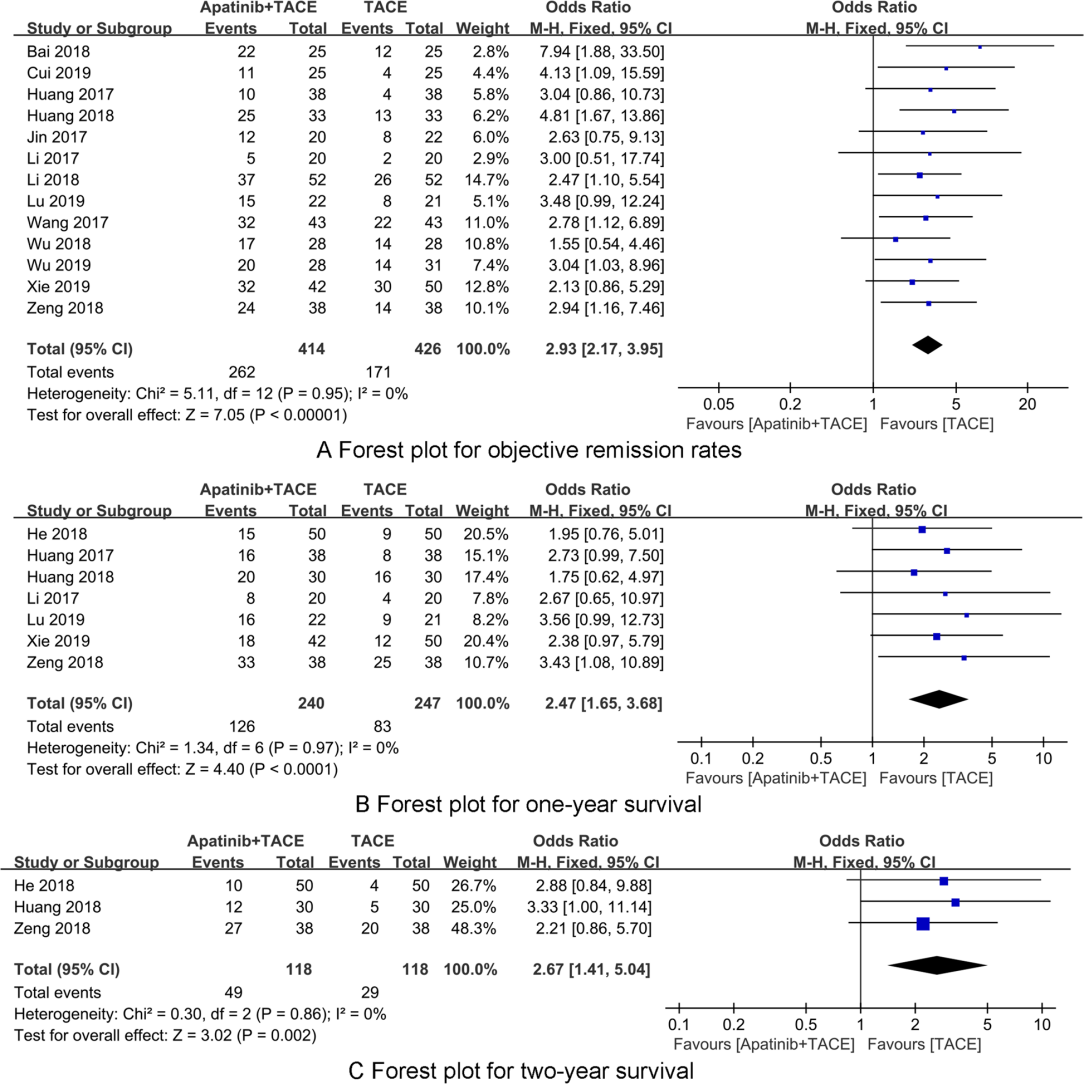
The forest plots for synthesized outcomes

Thirteen RCTs [22, 24–35] reported the objective remission rate. There was no heterogeneity among the included 13 RCTs (*P* = 0.92, *I*^2^ = 0%), and a fixed-effects model was used for synthesized analysis. The objective remission rate of the Apatinib + TACE group was significantly higher than that of the TACE group, and the difference was statistically significant (OR = 2.93, 95% CI 2.17–3.95, *P* < 0.001) (Fig. 4A). As presented in Table 2, subgroup analyses of the objective remission rate indicated that Apatinib + TACE treatment was beneficial to improve the objective remission rate both when Apatinib dose ≥ 600 or < 600 mg/day (all *p* < 0.001).

**Table 2:** Subgroup analyses of the objective remission rate and one-year survival based on the Apatinib dose

Outcomes	Subgroups	Number of included RCTs	Heterogeneity(I^2^)	Model	OR	95%CI	*P*
Objective remission rat	Apatinib dose≥600 mg/d	3	0%	Fixed	2.44	1.97~2.81	<0.001
	Apatinib dose<600 mg/d	10	0%	Fixed	2.91	2.53~3.67	<0.001
One-year survival	Apatinib dose≥600 mg/d	2	0%	Fixed	2.69	2.04~2.89	<0.001
	Apatinib dose<600 mg/d	5	16%	Fixed	2.17	1.89~2.55	<0.001

### One-year survival

Seven RCTs [23, 25, 28, 30–32, 34] reported the 1-year survival. There was no heterogeneity among the included 7 RCTs (*P* = 0.97, *I*^2^ = 0%), and a fixed-effects model was used for synthesized analysis. The 1-year survival of the Apatinib + TACE group was significantly higher than that of the TACE group, and the difference was statistically significant (OR = 2.47, 95% CI 1.65–3.68, *P* < 0.001) (Fig. 4B). As presented in Table 2, subgroup analyses of the 1-year survival indicated that Apatinib + TACE treatment was beneficial to improve the 1-year survival both when Apatinib dose ≥ 600 or < 600 mg/day (all *p* < 0.001).

### Two-year survival

Three RCTs [23, 25, 31] reported the 2-year survival. There was no heterogeneity among the included 3 RCTs (*P* = 0.86, *I*^2^ = 0%), and a fixed-effects model was used for synthesized analysis. The 2-year survival of the Apatinib + TACE group was significantly higher than that of the TACE group, and the difference was statistically significant (OR = 2.67, 95% CI 1.41–5.04, *P* = 0.002) (Fig. 4C).

### The incidence of myelosuppression

**Fig. 5:**
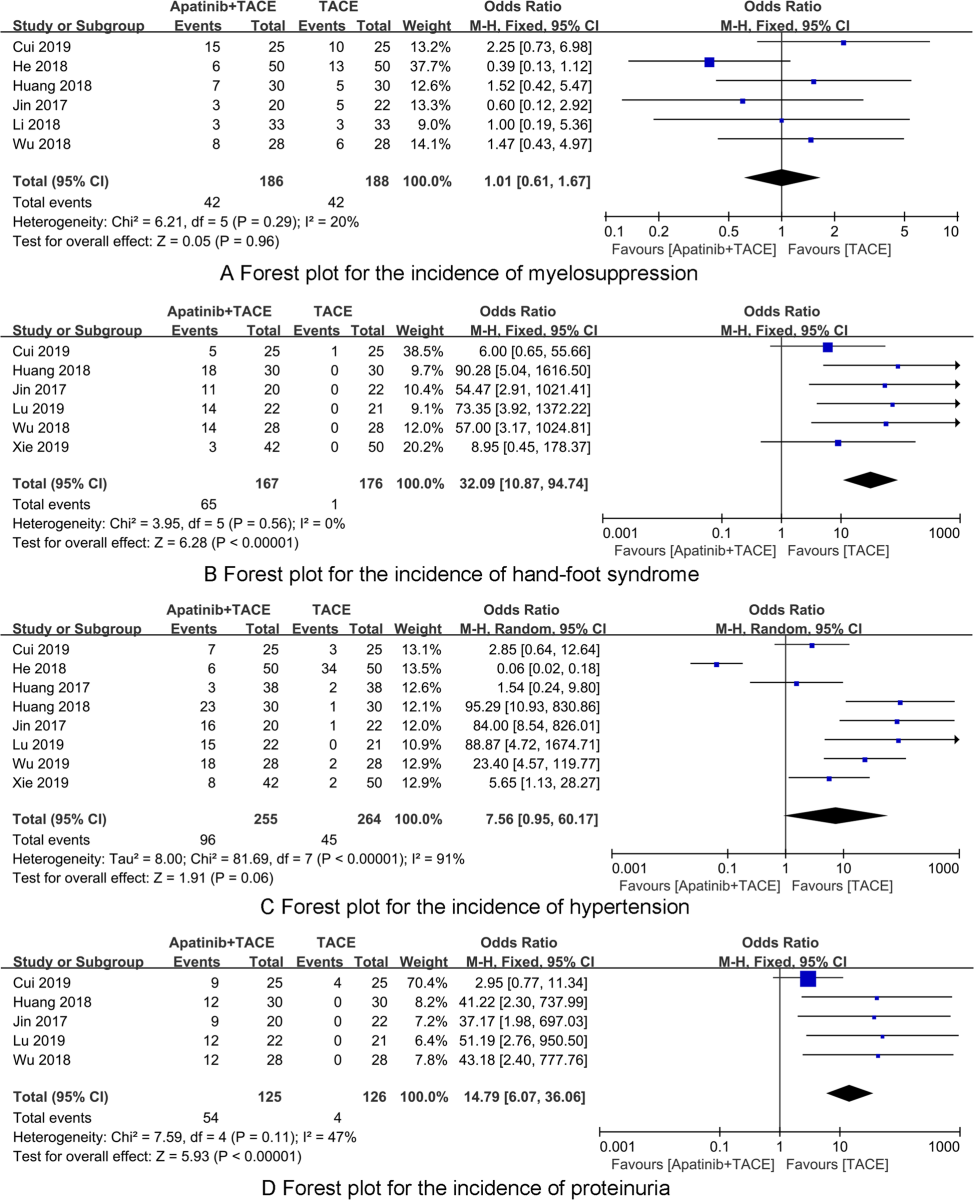
The forest plots for synthesized outcomes

Six RCTs [23, 26, 27, 31, 33, 35] reported the incidence of myelosuppression. There was no heterogeneity among the included 6 RCTs (*P* = 0.86, *I*^2^ = 0%), and a fixed-effects model was used for synthesized analysis. There was no significant difference in the incidence of myelosuppression between Apatinib + TACE and TACE group (OR = 1.01, 95% CI 0.61–1.67, *P* = 0.96) (Fig. 5A).

### The incidence of hand-foot syndrome

Six RCTs [26, 27, 30, 31, 33, 34] reported the incidence of hand-foot syndrome. There was no heterogeneity among the included 6 RCTs (*P* = 0.56, *I*^2^ = 0%), and a fixed-effects model was used for synthesized analysis. The incidence of hand-foot syndrome of the Apatinib + TACE group was significantly higher than that of the TACE group, and the difference was statistically significant (OR = 32.09, 95% CI 10.87–94.74, *P* < 0.001) (Fig. 5B).

### The incidence of hypertension

Eight RCTs [23, 24, 26, 28, 30, 31, 33, 34] reported the incidence of hypertension. There was heterogeneity among the included 8 RCTs (*P* < 0.001, *I*^2^ = 91%), and a random-effects model was used for synthesized analysis. There was no significant difference in the incidence of hypertension between Apatinib + TACE and TACE group (OR = 7.56, 95% CI 0.95–1.67, *P* = 60.17) (Fig. 5C).

### The incidence of proteinuria

Five RCTs [26, 27, 31, 33, 34] reported the incidence of proteinuria. There was no heterogeneity among the included 5 RCTs (*P* = 0.56, *I*^2^ = 11%), and a fixed-effects model was used for synthesized analysis. The incidence of proteinuria of the Apatinib + TACE group was significantly higher than that of the TACE group, and the difference was statistically significant (OR = 14.79, 95% CI 6.07–36.06, *P* < 0.001) (Fig. 5D).

### Publication bias

**Fig. 6:**
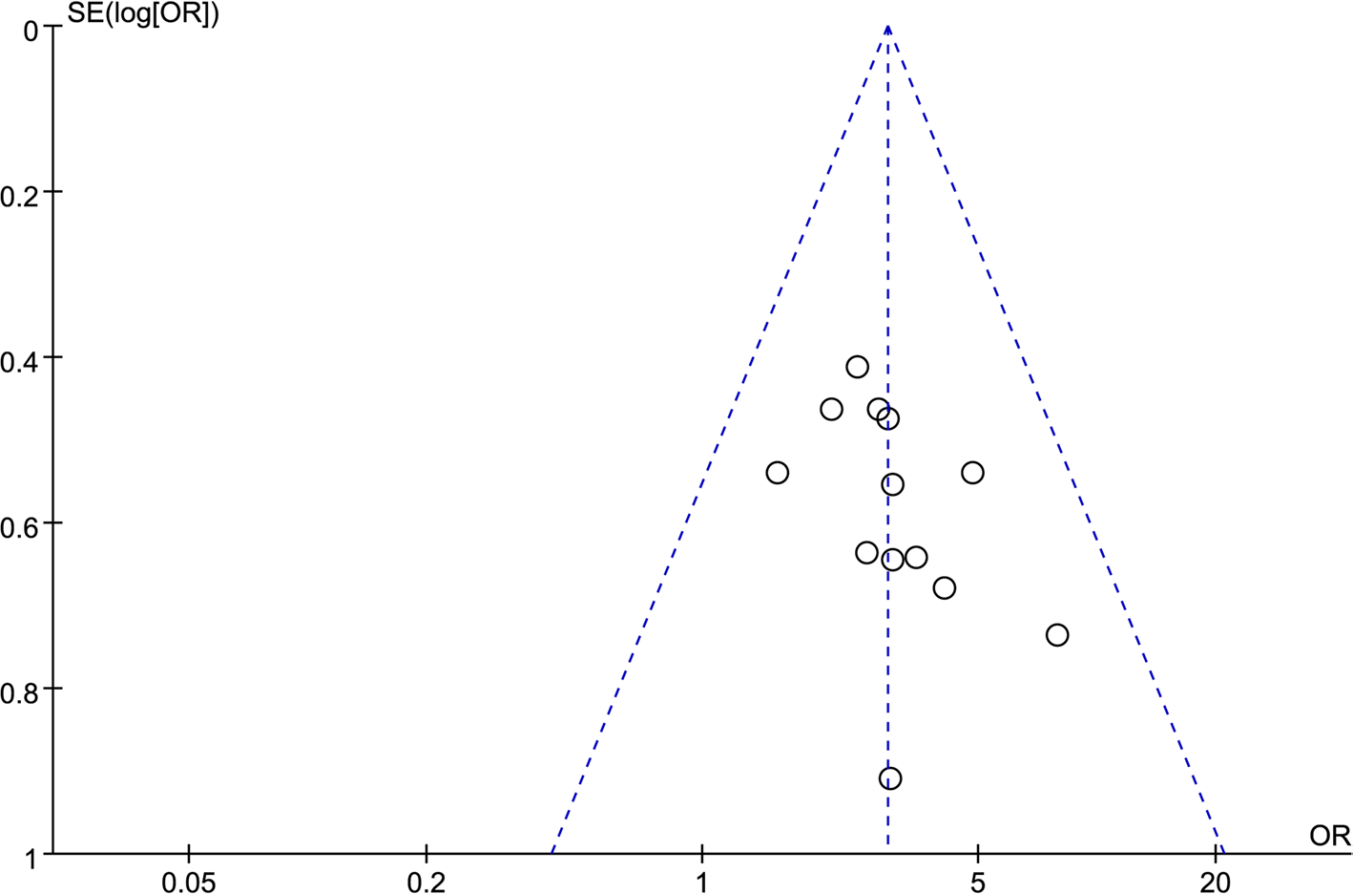
Funnel plot for the objective remission rates

We attempted to evaluate publication bias by using a funnel plot if 10 or more RCTs were included in an outcome meta-analysis. The funnel plot of objective remission rate (see Fig. 6) indicated that the scattered points were evenly and symmetrically distributed, suggesting that there was no publication bias.

Sensitivity analyses, which investigate the influence of 1 study on the overall risk estimate by removing study one by one, suggested that the overall risk estimates were not substantially changed by any single study. Besides, for the inconsistent result of He 2018 in the incidence of hypertension, it might be associated to the age differences between groups, the age of patients in the TACE group was significantly higher than that of Apatinib + TACE group (55.37 ± 10.33 vs. 52.14 ± 9.17).

## Discussions

With 14 RCTs included, this present meta-analysis has found that the combined use of Apatinib and TACE can significantly improve the objective remission rate, 1-year, and 2-year survival of patients with hepatocellular carcinoma compared with TACE treatment alone; yet, the combined use of Apatinib and TACE may also increase the incidence of hand-foot syndrome and proteinuria, and there was no significant difference in the incidence of myelosuppression and hypertension. However, all blinding information are most commonly under-reported, which may lead to result biases. Therefore, the results of this present meta-analysis should be treated with cautions, and future RCTs with rigorous design and longer follow-up are warranted.

Angiogenesis is closely associated with the occurrence, development, and metastasis of malignant tumors. Tumor cells can produce a variety of molecules to induce angiogenesis, the new blood vessels can provide nutrients necessary for tumor growth and excrete metabolites [50, 51]. Additionally, tumor cells are mainly transferred to other parts of the body through vascular dissemination [18]. Therefore, inhibiting tumor angiogenesis can inhibit tumor cell growth and metastasis. Previous studies [41, 52] has shown that tumor angiogenesis is a dynamic process with multi-factors participation. The signal cascade mediated by VEGFR-2 is the key regulatory pathway, which can regulate the proliferation, migration, survival and permeability of vascular endothelial cells [53]. It is been reported [54] that Apatinib can inhibit the proliferation of a variety of hepatocellular carcinoma cells, and the inhibitory effect is positively correlated with the expression of VEGFR-2. Apatinib can also affect the expression of cell cycle regulating point proteins, which in turn changes the cell cycle.

Apatinib is also an anti-angiogenesis molecular targeted drug, which by highly specific inhibition of the activity of the VEGFR-2 tyrosine kinase pathway, while blocking the signal transduction pathway after VEGF and its receptor are combined, it has been used for second-line treatment of gastric cancer [55]. It is been found that 60 μg/ml is the optimal concentration of Apatinib for radiotherapy in gastric cancer cells [56]. Apatinib combined with radiotherapy can reduce the negative effects of radiotherapy and reduce cancer-associated mortality [57]. However, there are also shortcomings in the biological treatment process. Skin inflammation, hypertension, gastrointestinal reactions, proteinuria, cytopenias, hand-foot syndrome, abdominal pain, and abdominal distension often occur during treatment, which can be alleviated by symptomatic supportive treatment [58]. It’s been reported that Apatinib is well tolerated and very effective in the treatment of advanced HCC, and it is beneficial in terms of objective remission rate and disease control rate [59]. Previous studies [60, 61] have shown that TACE combined with Apatinib has a better mid- and long-term efficacy for the treatment of advanced hepatocellular carcinoma, and has a certain degree of safety.

This study does have the following limitations. Firstly, we only searched for the reports published in the language of English and Chinese, and the RCTs included in this meta-analysis are all from China, and the results of the study may have regional and ethics biases. Secondly, the most included studies did not mention specific random allocation methods, and most of them did not mention blinding design and allocation concealment. We have included heterogeneous regimens based on the Apatinib dose to analyze the effects of Apatinib and TACE treatments. Thirdly, the sample size is not large enough, and it may be underpower to detect the potential difference between groups. It is necessary to conduct higher-quality researches with rigorous design to further evaluate the role of Apatinib in the treatment of hepatocellular carcinoma in the future.

## Conclusions

To sum up, the combination of Apatinib and TACE can improve the objective remission rate and 1-year and 2-year survival of patients with hepatocellular carcinoma. In terms of safety, the use of Apatinib may have higher risk of hypertension, hand-foot syndrome, and proteinuria. The occurrence of such adverse reactions should be considered in clinical applications. Still, the dosage and duration of Apatinib in the treatment of hepatocellular carcinoma has not yet been unified. It is still too early to try to make certain assumptions. In the future, large-scale, high-quality clinical studies from the perspective of dosage and different populations are needed to further elucidate the effects and safety of Apatinib in the treatment of hepatocellular carcinoma.

## Supplementary Information

**Additional file 1: Supplementary Table 1**. Search Strategy for Each Database.

## Data Availability

All data generated or analyzed during this study are included in this published article.

## Abbreviations

TACE: Transcatheter arterial chemoembolization
VEGF: Vascular endothelial growth factor
RCT: Randomized controlled trial
CENTRAL: Cochrane Central Register of Controlled Trials
