# Supplementary material for: The efficacy and safety of Apatinib combined with TACE in the treatment of hepatocellular carcinoma: a meta-analysis
Source: World J Surg Oncol. 2022 Mar 4;20:69. doi: 10.1186/s12957-021-02451-8 (PMC8897864; doi:10.1186/s12957-021-02451-8)
Supplement: Supplementary file 1 — Additional file 1: Supplementary Table 1. Search Strategy for Each Database. [file 12957_2021_2451_MOESM1_ESM.docx]

Supplementary Table 1. Search Strategy for Each Database

| **Database** | **Search strategy** |
| --- | --- |
| Pubmed | #1 " Neoplasms "[MeSH Terms] OR " Hepatic"[All Fields] OR" Hepatocellular" [MeSH Terms]  #2 " Apatinib"[All Fields]  #3 " Transcatheter arterial chemoembolization"[MeSH Terms] OR " TACE "[All Fields] OR " Hepatic arterial chemoembolization"[All Fields]  #4 systematic[sb] OR Meta-Analysis[ptyp]  #5 #1 or #2  #6 #1and #3 and #5  #7 #1 and #4 and #6 |
| Embase | #1 ' Neoplasms'/exp OR ' Hepatocellular'  #2 ' Apatinib '/exp OR ' Apatinib '  #3 ' Transcatheter arterial chemoembolization '/exp OR TACE  #4 [cochrane review]/lim OR [systematic review]/lim OR [meta analysis]/lim  #5 #1 or #2  #6 #3 and 5  #7 #4 and #6 |
| Cochrane library | #1 Hepatocellular:ti,ab,kw (Word variations have been searched)  #2 Apatinib:ti,ab,kw (Word variations have been searched)  #3 Transcatheter arterial chemoembolization:ti,ab,kw (Word variations have been searched)  #4 #1 or #2  #5 #3 and #4 (restricted as Cochrane Reviews or other reviews) |
| China Biomedical Literature Database | #1 " 肝癌 "[关键词] OR "肝"[全部] OR"癌症" [关键词]  #2 "阿帕替尼"[关键词]  #3 "经导管动脉化疗栓塞"[关键词] OR " TACE "[全部]  #4 systematic[关键词] OR Meta-Analysis[关键词]  #5 #1 or #2  #6 #1and #3 and #5  #7 #1 and #4 and #6 |
| China Knowledge Network | #1 " 肝癌 "[关键词] OR "肝"[全部] OR"癌症" [关键词]  #2 "阿帕替尼"[关键词]  #3 "经导管动脉化疗栓塞"[关键词] OR " TACE "[全部]  #4 systematic[关键词] OR Meta-Analysis[关键词]  #5 #1 or #2  #6 #1and #3 and #5  #7 #1 and #4 and #6 |
| Wanfang Database | #1 " 肝癌 "[关键词] OR "肝"[全部] OR"癌症" [关键词]  #2 "阿帕替尼"[关键词]  #3 "经导管动脉化疗栓塞"[关键词] OR " TACE "[全部]  #4 systematic[关键词] OR Meta-Analysis[关键词]  #5 #1 or #2  #6 #1and #3 and #5  #7 #1 and #4 and #6 |
| Weipu Chinese Science and Technology Journal Database | #1 " 肝癌 "[关键词] OR "肝"[全部] OR"癌症" [关键词]  #2 "阿帕替尼"[关键词]  #3 "经导管动脉化疗栓塞"[关键词] OR " TACE "[全部]  #4 systematic[关键词] OR Meta-Analysis[关键词]  #5 #1 or #2  #6 #1and #3 and #5  #7 #1 and #4 and #6 |
